# Sex, Age and Stature Affects Neck Biomechanical Responses in Frontal and Rear Impacts Assessed Using Finite Element Head and Neck Models

**DOI:** 10.3389/fbioe.2021.681134

**Published:** 2021-09-21

**Authors:** M. A Corrales, D. S Cronin

**Affiliations:** Department of MME, University of Waterloo, Waterloo, ON, Canada

**Keywords:** neck biomechanical response, age effects, sex effects, finite element model, frontal impact, stature effects, size effects, rear impact

## Abstract

The increased incidence of injury demonstrated in epidemiological data for the elderly population, and females compared to males, has not been fully understood in the context of the biomechanical response to impact. A contributing factor to these differences in injury risk could be the variation in geometry between young and aged persons and between males and females. In this study, a new methodology, coupling a CAD and a repositioning software, was developed to reposture an existing Finite element neck while retaining a high level of mesh quality. A 5th percentile female aged neck model (F05_75YO_) and a 50th percentile male aged neck model (M50_75YO_) were developed from existing young (F05_26YO_ and M50_26YO_) neck models (Global Human Body Models Consortium v5.1). The aged neck models included an increased cervical lordosis and an increase in the facet joint angles, as reported in the literature. The young and the aged models were simulated in frontal (2, 8, and 15 g) and rear (3, 7, and 10 g) impacts. The responses were compared using head and relative facet joint kinematics, and nominal intervertebral disc shear strain. In general, the aged models predicted higher tissue deformations, although the head kinematics were similar for all models. In the frontal impact, only the M50_75YO_ model predicted hard tissue failure, attributed to the combined effect of the more anteriorly located head with age, when compared to the M50_26YO_, and greater neck length relative to the female models. In the rear impacts, the F05_75YO_ model predicted higher relative facet joint shear compared to the F05_26YO_, and higher relative facet joint rotation and nominal intervertebral disc strain compared to the M50_75YO_. When comparing the male models, the relative facet joint kinematics predicted by the M50_26YO_ and M50_75YO_ were similar. The contrast in response between the male and female models in the rear impacts was attributed to the higher lordosis and facet angle in females compared to males. Epidemiological data reported that females were more likely to sustain Whiplash Associated Disorders in rear impacts compared to males, and that injury risk increases with age, in agreement with the findings in the present study. This study demonstrated that, although the increased lordosis and facet angle did not affect the head kinematics, changes at the tissue level were considerable (e.g., 26% higher relative facet shear in the female neck compared to the male, for rear impact) and relatable to the epidemiology. Future work will investigate tissue damage and failure through the incorporation of aged material properties and muscle activation.

## Introduction

The elderly population has been identified to have an increased incidence of injury, compared to a young population, under similar loading in vehicular crashes (Lomoschitz et al., 2002; Kahane, 2013). The increased injury risk has been attributed, in part, to the change in posture associated with age (Park et al., 2016a). Specifically, within the neck, neck pain prevalence in the elderly (70–74 years old (YO)), is higher than in the younger population (Safiri et al., 2020) while vehicular crashes have been identified as one of the main causes of neck injuries (Umana et al., 2018). It has been found that the elderly exhibit increased lordosis of the cervical spine (D. Klinich et al., 2012) due to the combined effect of the increased kyphosis of the thoracic spine (Drzał-Grabiec et al., 2012) and orientation of the head to maintain the infraorbital-tragion line orientation. In addition to the increased lordosis in the neck with age, the cervical spine undergoes other morphological changes, such as an increase in facet angle (Parenteau et al., 2014). The isolated effect of the posture and morphological changes associated with increasing age on the tissue response has not been fully understood (Schoell et al., 2015) and has not been investigated in the neck region where some of the largest posture changes occur. In addition, it has been shown that small stature female occupants demonstrate a higher incidence of injury in car crash events (Bose et al., 2011) when compared to mid-size males. It has also been reported that females have a higher risk of Whiplash Associated Disorders (WAD) than males (Carlsson, 2012) in rear impacts. These outcomes are potentially related to the geometrical features (e.g., cervical lordosis, facet angle and size) of females, compared to males, and how they interact with the vehicle seat and safety systems (Kullgren et al., 2013).

Injury to the neck can occur as a catastrophic failure of tissues (e.g., ligament rupture and hard tissue failure) or sub-catastrophic tissue distraction that can lead to pain response (i.e. WAD), often associated with low severity impacts (Yang, 2018). Among the tissues associated with WAD in the neck, the sub-catastrophic collagenous fiber realignment of the capsular ligament (CL) and tears in the anterior annulus fibrosus of the intervertebral disc (IVD) has been associated with pain response (Yoganandan et al., 2001; Cavanaugh, 2006; Quinn and Winkelstein, 2007; Curatolo et al., 2011). In addition to direct tissue response (e.g. CL deformation), it has been proposed that relative facet joint kinematics (FJK) can be used to infer injury or pain response in the facet joint (Stemper et al., 2011b); for example, the relative displacement of the superior facet along the plane of the inferior facet surface represents shear displacement of the facet joint. Large shear displacements of the facet joint could be associated with an injurious capsular ligament strain. Similarly, nominal IVD shear strain has been used in experimental and computational studies to infer the likelihood of injury based on tissue kinematics (Panjabi et al., 2004; Fice and Cronin, 2012). Therefore, differences in catastrophic tissue failure, sub-catastrophic tissue strain and relative facet joint kinematics between young and aged subjects are of interest. Importantly, the quantification of the differences in the kinematic response and soft tissue response between males and females and the effect of the ageing process is limited.

With respect to the ageing geometrical changes, it has been shown that the cervical tissue morphology (Parenteau et al., 2014) and overall neck posture (Reed and Jones, 2017) change with age. Parenteau measured cervical facet angle, vertebral body depth and maximum spinal canal diameter of 251 CT scans of male subjects with an age range from 18 to 80 years old (YO). The sample was then divided into four age groups (18–29, 30–44, 45–59, and 60+), and it was found that the 60 + group had an increased facet angle (*p* < 0.0001), increased vertebral body depth at the C4, C5, and C6 levels (*p* < 0.0001), and a decreased spinal canal radius (*p* < 0.1) with respect to the 18–29 YO age group. In a separate study, Reed and Jones developed a cervical spine posture predictor (CSP) for a driving position based on gender, stature, seated stature, and age. A total of 177 seated position subjects from 18 to 74 YO were radiographed in neutral posture, maximum extension, and maximum flexion (Snyder et al., 1975) and digitized (Desantis Klinich et al., 2004) to serve as the database of the CSP. An increased lordosis in the cervical spine in the elderly population was identified, which was in agreement with previous studies (Boyle et al., 2002; Klinich et al., 2012). Both studies demonstrated an increased vertebral body depth and an increased facet angle with increasing age. Importantly, both studies suggest that the females had a higher increase in cervical lordosis and facet angle with age than the males. Another study (Park et al., 2016b) measured the posture in a driving-like environment (seated looking forward with hands on the steering wheel) of 46 male subjects with an age range of 21–95 YO. A general full-body posture predictor (FBP) in a driving position as a function of age, body mass index, stature, seated stature, seat height and seatback angle was developed. The predictor outputs coordinate points representing the center of the eye, tragion, C7/T1 joint, T12/L1 joint, mid-hip joint, knee joint and ankle joint. Regarding age, the study concluded that the aged occupants have a more anteriorly located head center of gravity than the young occupants, attributed to the increased thoracic kyphosis and cervical lordosis. Regarding the geometrical differences between males and females, the circumference of the female cervical spine relative to the length of the neck is smaller, as is the vertebral body sizes, and it has smaller muscle cross-sectional area for stature matched subjects (Vasavada et al., 2008; Stemper et al., 2011a).

**FIGURE 1:**
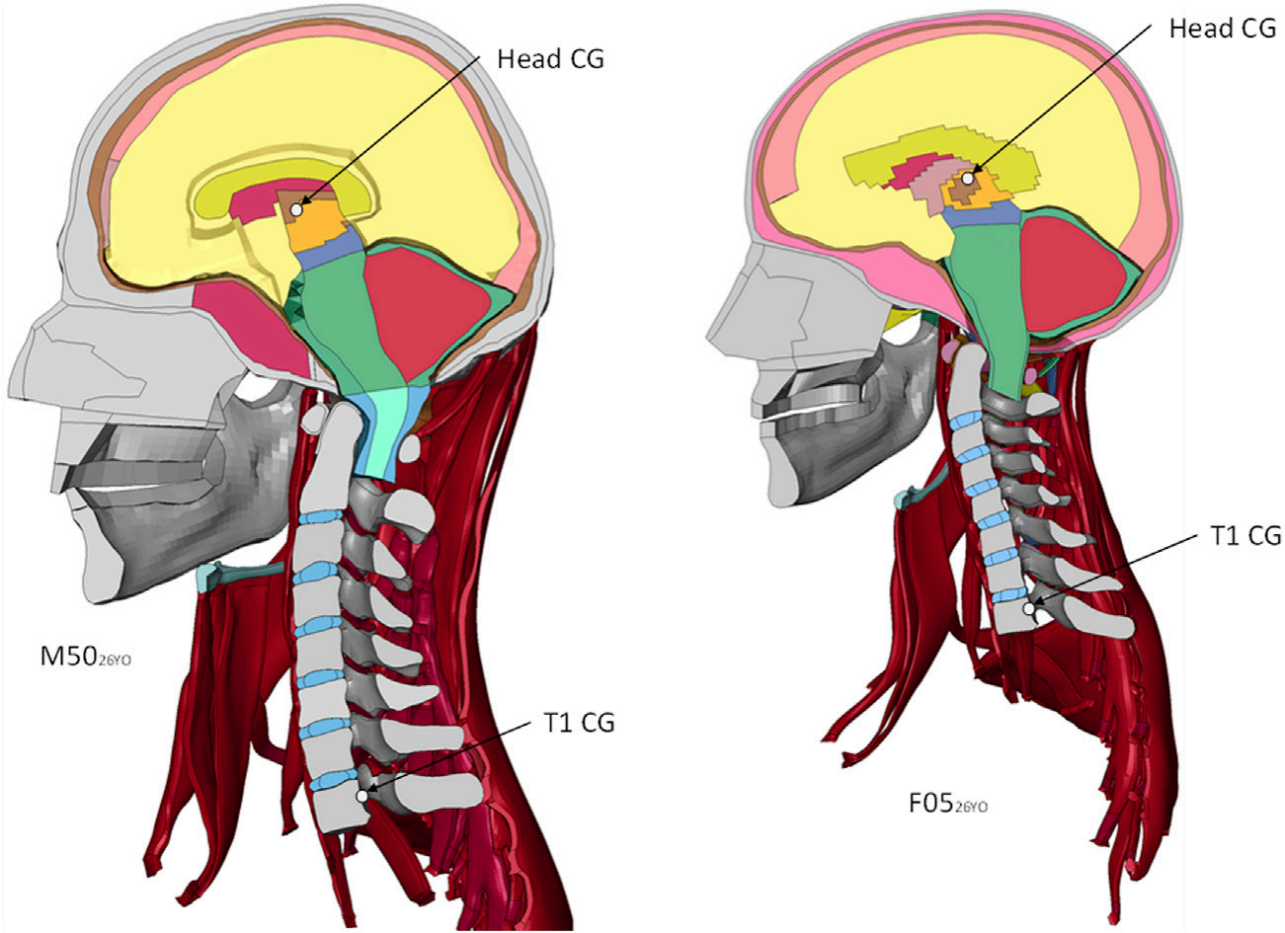
Sagittal plane view of the detailed GHBMC Neck and Head models, showing the head center of gravity (CG) and the T1. Boundary conditions are applied at the T1.

Finite element (FE) models are commonly used to assess the effect of isolated factors in the mechanical response of a system, such as geometrical changes. Human body models (HBM) are widely used to increase the understanding of kinematics in impact events, such as vehicle crashes and injury risk. Two contemporary HBM include the Global Human Body Models Consortium (GHBMC) average stature male (M50_26YO_) (GHBMC M50-O v5.1) and small stature female (F05_26YO_) (GHBMC F05-O v5.1) (Figure 1). The geometry of the existing (young) models was based on magnetic resonance imaging scans and computerized tomography scans of a 26 YO male volunteer representative of a 50th percentile male (Gayzik et al., 2011) and a 26 YO female volunteer representative of a 5th percentile female (Davis et al., 2014). (Gayzik et al., 2011; Davis et al., 2014) A recent study (Barker et al., 2017) validated the M50_26YO_ neck model at the motion segment level against a wide range of experimental data in quasi-static and dynamic traumatic loading. At the full neck level, the model was validated (Barker and Cronin, 2021) in rear impacts using cadaveric full neck experimental data and in frontal and lateral impacts using human volunteer data. The active muscle activation scheme of the M50_26YO_ and F05_26YO_ was developed previously using volunteer data (Correia et al., 2020). The open-loop co-contraction muscle activation scheme (Correia et al., 2020) was designed to contract the neck muscles while maintaining the head in a neutral posture. The GHBMC neck model was objectively compared to the experimental data using the cross-correlation and corridor method (Correia et al., 2020; Barker and Cronin, 2021) with good cross-correlation ratings. The GHBMC M50_26YO_ and F05_26YO_ models include equivalent-plastic-strain-based element erosion criteria to model cortical and trabecular bone fracture. The cortical material model (Khor et al., 2018) was validated in a femur model under axial rotation and three-point bending. In the cervical spine, the cortical and trabecular bone models with bone fracture included were validated (Khor et al., 2017)in a C5-C6-C7 functional spinal unit under axial and eccentric compression with good agreement at the kinematic level (force-displacement response).

However, detailed HBMs have been developed in a limited number of positions (e.g., driving posture and pedestrian). Simplified models can be repositioned with simple transformation tools in pre-processor packages (e.g. LS-PrePost). For example, Frechede et al., 2006 investigated the effect of neck curvature in a simplified head and neck FE model by transforming the vertebra to achieve three postures (lordotic, straight and kyphotic) defined using Cobb angles. However, detailed models are challenging to reposition while retaining the mesh quality in the soft tissue (Janak et al., 2018). A recently released repositioning software (PIPER), developed to reposition and morph detailed HBM, without retaining the resultant stress state (Beillas et al., 2015), allows researchers to precisely reposition detailed FE models while retaining mesh quality (Janak et al., 2018).

There were two main objectives of this study. First, to investigate the effect of geometrical factors associated with the aging process on tissue-level response; therefore, the cervical spine lordosis and facet joint angle were modified while the material properties and the muscle activation scheme were held constant. The second objective was to compare the tissue-level response of the young and aged average stature male models to the young and aged small stature female models under frontal and rear impacts of various severities.

## Materials and Methods

In the present study, two existing young neck models (Figure 1) were extracted from contemporary detailed full HBMs M50-O v5.1 (M50_26YO_) and F05-O v5.1 (F05_26YO_). The M50_26YO_ and F05_26YO_ models were repostured to represent the posture of an average 75 YO subject, and the facet pillars were morphed to represent the facet angle change associated with age. Four models were evaluated in the present study; the existing M50_26YO_ and F05_26YO_ and the newly developed aged models (M50_75YO_ and F05_75YO_) to assess the effect of age and sex differences on model response and the potential for injury. Head kinematics, FJK, and CL and IVD strain of the M50_75YO_ and F05_75YO_ were monitored and compared to those of the M50_26YO_ and F05_26YO_ models in frontal (2, 8, and 15 g) and rear (3, 7, and 10 g) impacts. The GHBMC HBMs are in the units of mm, ms and kg.

### Posture Definition

**FIGURE 2:**
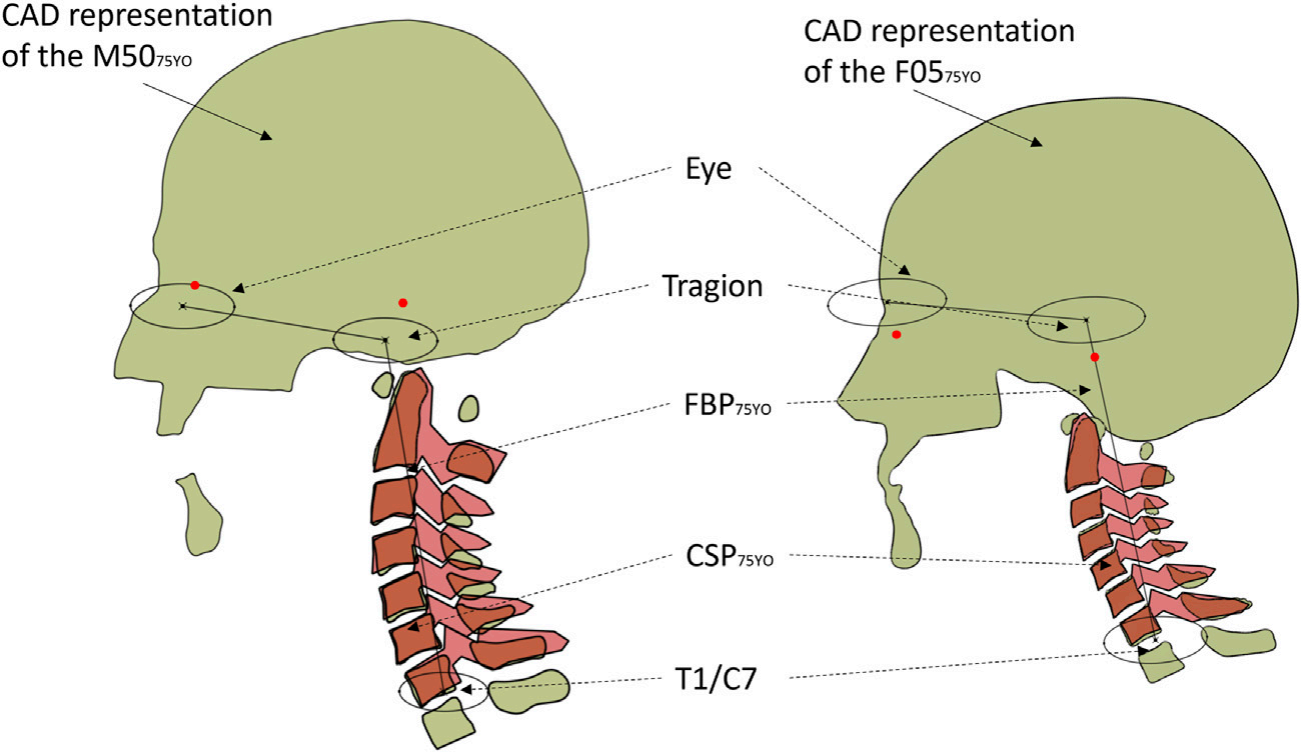
The aged GHBMC CAD representation (light green) overlapped with the C-Spine predictor (red) for the aged M50_75YO_ (left) and F05_75YO_ (right). Red dots show the infraorbital and tragion of the head and neck models compared to the full-body predictor (black lines and ellipses).

A novel approach introduced in the current study is the use of CAD to improve the ease of comparing the model to literature data and to incorporate literature data to the definition of the reposturing targets in order to reduce reposturing time by 30–50%. A CAD (CATIA V5, Dassault systems) representation of the FE cervical spine model was developed (Figure 2). First, the posture of the M50_26YO_ model was compared to the CSP (Reed and Jones, 2017) and FBP (Park et al., 2016b) data. The anthropometrics corresponding to the M50_26YO_ model were used as input for the CSP and FBP models (1749 mm standing stature, 26 years old and 0.53 for the ratio standing/seated stature). It was found that the subject-specific M50_26YO_ model had a longer neck (10.8%) than the single posture reported by the CSP neck length for the given stature, age and seated height ratio of the M50_26YO_. A set of anthropometrics that match the posture and the neck length of the M50_26YO_ model were found by increasing the stature to 1846 mm (5.5% increase in height with respect to the M50_26YO_).

To define the aged posture, the age in the CSP was changed from 26 YO (1846 mm standing stature, 26 YO and 0.53 for the ratio standing/seated stature) to 75 YO (1846 mm standing stature, 75 YO and 0.53 for the ratio standing/seated stature). The change in stature with increasing age has been reported to be 2–4 cm over the life course (Fernihough and McGovern, 2015) and was excluded from this study. The vertebral bodies in the CAD assembly representing the M50_26YO_ model were translated and rotated accordingly to the aged posture predicted by the CSP (Reed and Jones, 2017) to define the M50_75YO_ posture. The superior endplate and the posterior edge of the vertebral body were prioritized over the inferior endplates when defining the aged posture. The aged posture was then compared to the FBP for posture validation (Park et al., 2016a). It was found that M50_75YO_ had a longer neck than the average population measured in the FBP, but the general posture was considered in agreement given the variability of the lumbar and thoracic regions. For each vertebra, three landmarks were extracted from the CAD assembly for the aged neck posture: the geometric center of the superior endplate and the most distal point of the posterior transverse processes. Those landmarks served as input for the reposturing. The same procedure was applied to the F05_26YO_ model to define the F05_75YO_ model posture. The curvature and length of the F05_26YO_ model agreed with both the CSP and the FBP. The specific locations of the landmarks used in the present study are included in the published F05 PIPER metadata (http://www.piper-project.eu).

Following the definition of the aged posture, the facet joint angles for the aged models were defined using the percentage of increase reported in the literature (Parenteau et al., 2014) at each segment level. Given that the intent of the present work was to develop the aged version of the existing subject-specific models, the relative percent increase in the facet angle from young to old was used to modify the facet angles from the young to old models. The females were reported to have an increase of 10.9% in the facet angle with age, whereas the males a 5.6% of increase when averaging all the segment levels (Parenteau et al., 2014).

### Repositioning and Morphing

The young neck models were repostured to the aged target posture using contemporary repositioning software (PIPER) (Beillas et al., 2015). The reposturing process required model-specific metadata (skin definition, hard tissue definition and landmarks) within the HBM to successfully achieve the target posture. The metadata required to reposture the neck region of the M50 and F05 models were developed in the present study. The F05 neck region metadata used in this study, along with full-body metadata, was made available to the community (http://www.piper-project.eu). Using the targets for an aged neck posture, the models were repositioned by moving the vertebrae to the desired location. After the target neck curvature was achieved, the facet pillars were morphed to achieve the target facet angle using PIPER. The behavior of the soft tissues was calculated by the PIPER software based on simplified material properties and the simulation engine “SOFA” (SOFA, National Institute for Research in Digital Science and Technology, France), another open-source package meant to simulate soft tissue behavior in clinical applications. The resultant stress-strain state after reposturing was not retained since the aged models were developed to be in a neutral posture for a specific age. All models in their respective neutral postures were assumed to be at a zero stress and strain state. After the target neck curvature was achieved, the facet pillars were morphed to increase the facet angle using PIPER. The PIPER engine calculates the position of the soft tissue during the repositioning simulation. Following the neck repositioning, the muscle, flesh, and skin meshes were smoothed using the transformation smoothing option (Janak et al., 2018) in PIPER. The mesh quality of the M50_75YO_ and F05_75YO_ models was assessed using the metrics and thresholds of the M50_26YO_ and F05_26YO_ models (including warpage <50°, aspect ratio <8, skew <70°, and Jacobian >0.4) and checked for penetrations. Static (50 ms with no boundary conditions) and dynamic (15 g frontal, 7 g lateral, and 7 g rear impacts for 235 ms) stability simulations ran to normal termination.

### Model Evaluation

**FIGURE 3:**
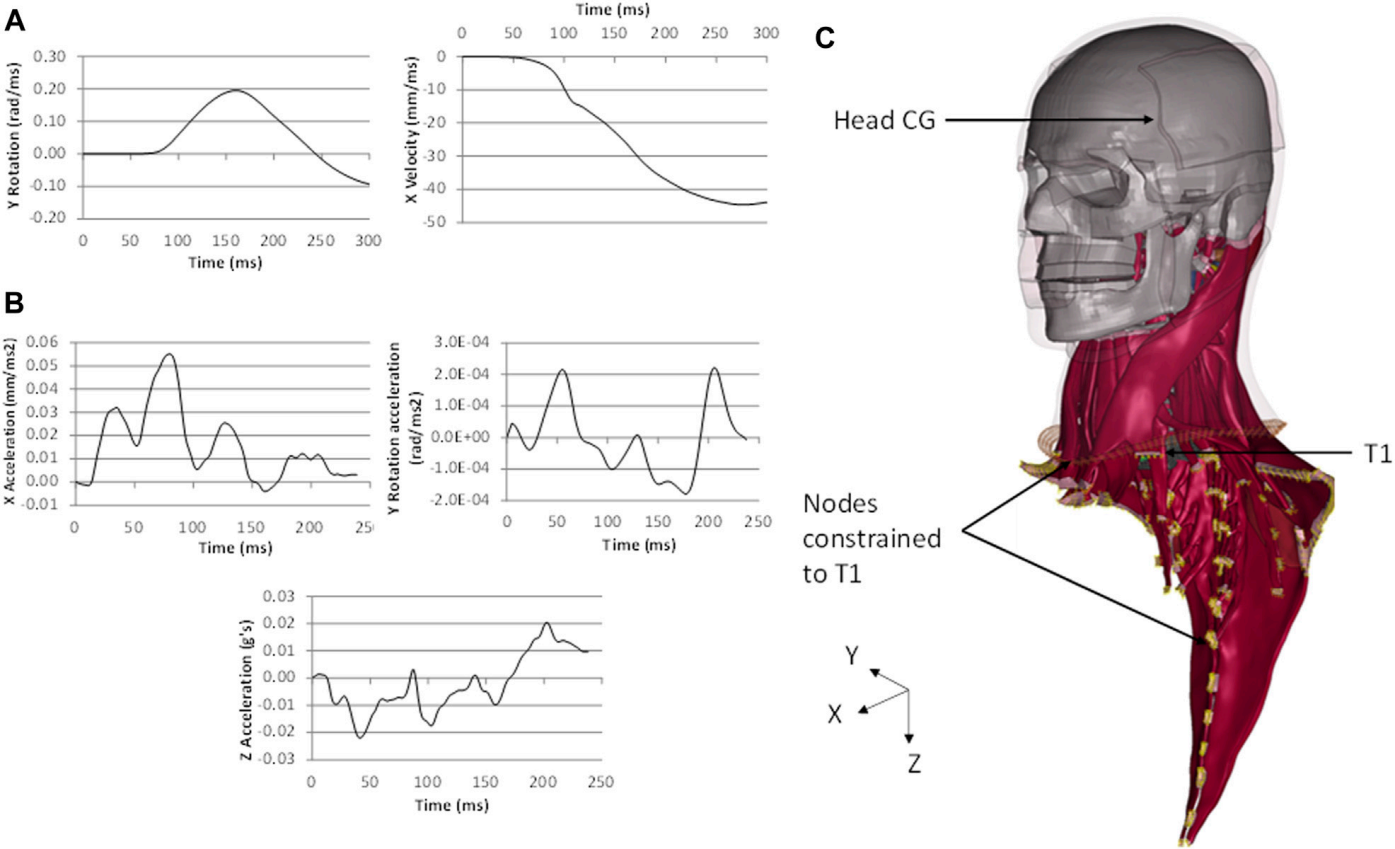
Boundary conditions of (**A**) the 8 g frontal impact and (**B**) 7 g rear impact applied to (**C**) the first thoracic vertebrae (T1) of the head and neck models (M50_26YO_ shown). Nodes constrained to T1 showed in yellow.

The four head and neck models were subjected to frontal (2, 8, and 15 g) and rear impact (3, 7, and 10 g) impacts using boundary conditions reported in the literature (Wismans et al., 1987; Deng et al., 2000) and developed for the GHBMC neck model (Barker and Cronin, 2021) (Figures 3A,B). The boundary conditions were applied to the first thoracic vertebra (T1). The nodes in the muscle insertions below T1, and the last layer of flesh and skin nodes were rigidly fixed to the T1. The rest of the model remained unconstrained (Figure 3C).

**FIGURE 4:**
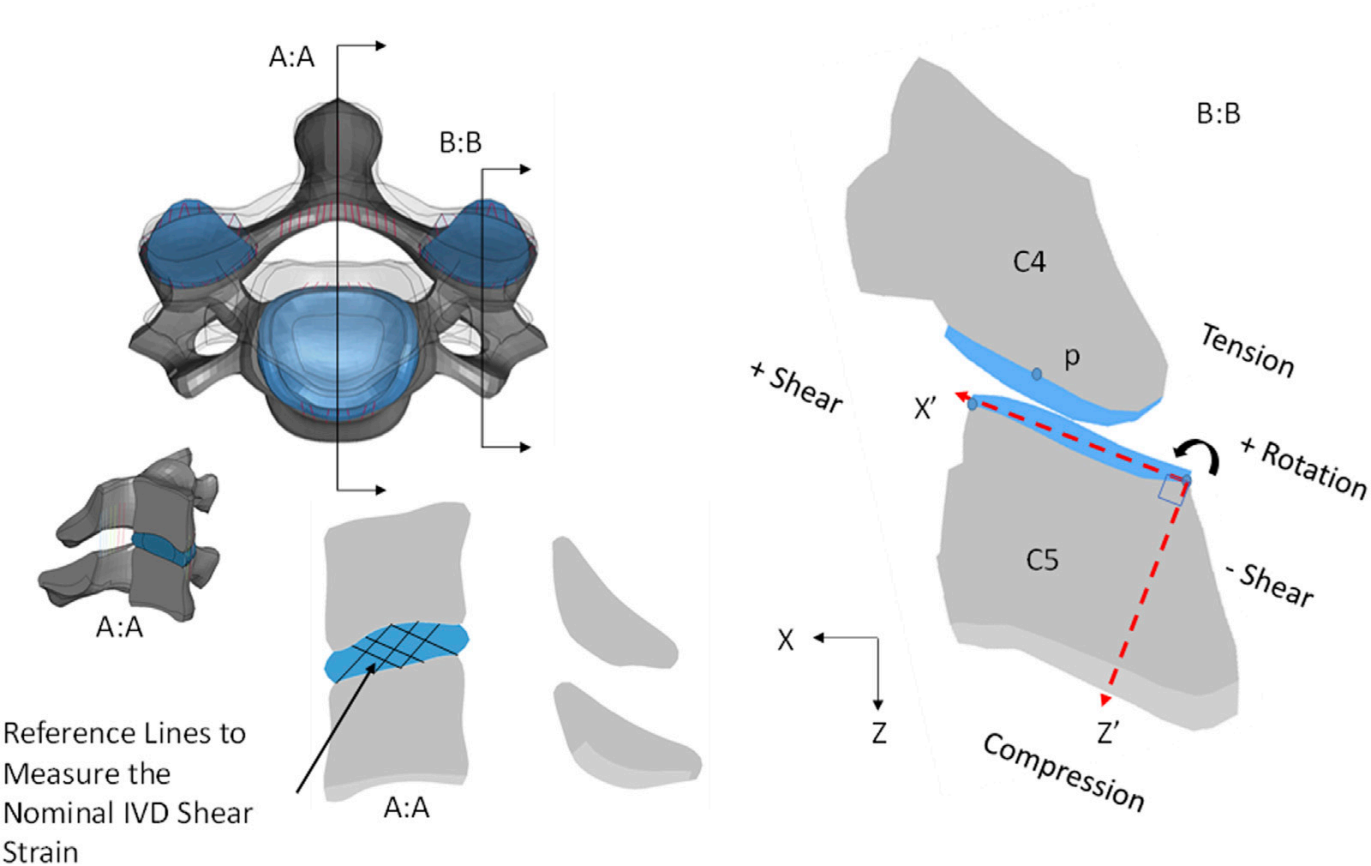
(**A)** Nominal IVD shear strain measured using the change in angle between reference lines as in previous computational and experimental studies (Panjabi et al., 2004; Fice and Cronin, 2012) and (**B**) relative facet joint kinematics (FJK) calculated as the displacement of the point “p” in the inferior facet of the vertebra with respect to a local coordinate system in the superior facet of the lower adjacent vertebra (M50_26YO_, C45 segment shown).

The models were assessed at three levels: head kinematics, relative FJK and nominal IVD shear strain. The head kinematics were extracted directly from the head CG of the model using a post-processor (LS-PrePost version 4.7.20). The head kinematic response of the young models was objectively compared to their aged counterparts using the cross-correlation method. The cross-correlation (CORA, pdb, Germany) is an objective method to compare the model response (e.g. aged model kinematic response) to a reference curve (e.g. young model kinematic response). The level of correlation is calculated as a value between 0 and 1, where 1 means perfect correlation and 0 means no correlation. The FJK were calculated as the displacements of the point “p” in the inferior facet of the vertebra (C2 to C7) with respect to a local coordinate system (X′, Z′) in the superior adjacent vertebra (Figure 4B), similar to experimental (Stemper et al., 2011b) and computational studies (Corrales and Cronin, 2021). The FJK rotation was defined as the change in angle between the X′ axis and a line passing through the local coordinate system origin and the point “p”. In the present study, FJK shear displacement was defined as the displacement of the point “p” along the X′ axis and the FJK compression defined as the displacement of the point “p” along the Z′ axis (Figure 4B). The nominal IVD shear strain was measured using the change in angle between reference lines formed by discrete points in the endplates of the adjacent vertebrae as reported in previous experimental (Panjabi et al., 2004) and computational (Fice et al., 2011) studies (Figure 4A). It should be noted that nominal IVD shear strain does not correspond to the strain in the tissue but rather the deformation of the IVD, based on the relative position of the vertebral bodies. In this study, it will be referred to as nominal IVD shear strain for consistency with the previous experimental and computational studies. The FJK and nominal IVD shear strain were calculated for each segment level (Supplementary Appendix B and C) and then averaged for clarity in the results section. In addition, the GHBMC neck model incorporates cortical and trabecular bone failure criteria (element erosion based on a critical effective plastic strain), ligament failure (displacement-based progressive element erosion), and IVD avulsion (tied interface criterion based on critical stress) (Barker et al., 2017; Barker and Cronin, 2021). Hard tissue failure (Khor et al., 2018), ligament failure and IVD avulsion (Barker et al., 2017) were monitored in the four models.

## Results

### Aged Posture and Comparison to Geometric Data

**FIGURE 5:**
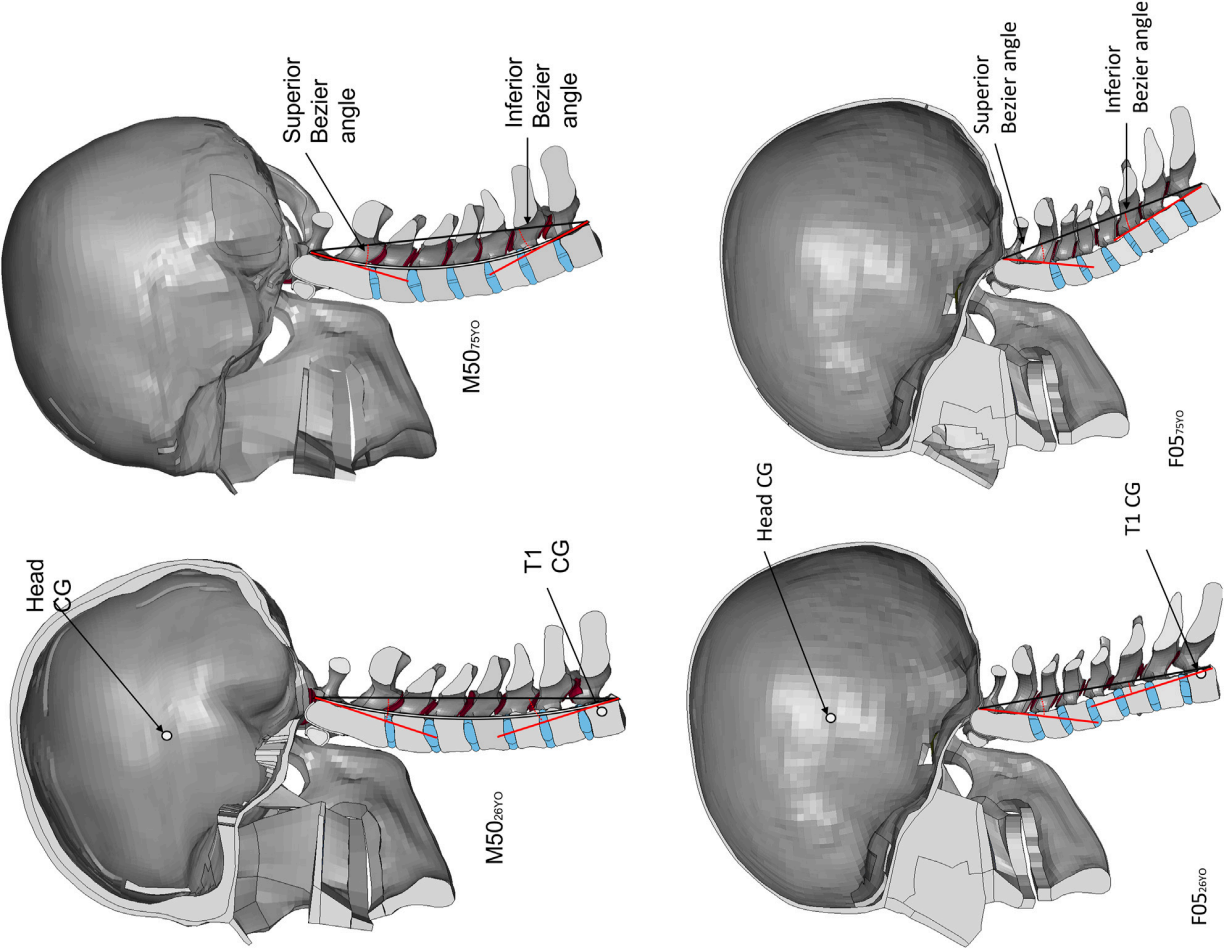
The M50_26YO_ and F05_26YO_ on the left and the newly developed 75 YO models, M50_75YO_ and F05_75YO_, on the right. Measurement of the Bezier angles illustrated in the models.

The final position of the M50_75YO_ and F05_75YO_ models hard tissues was within 0.9 microns of the target positions, measured at the corners of the vertebral body. The location of the tragion and eye of the models were outside one standard deviation of the full-body predictions (Park et al., 2016b), attributed to the thoracic length and curvature of the subject-specific models. Importantly, the head orientation of the young and aged models matched the predicted head orientation of the full-body predictions in a driving position. The Bezier angles (Figure 5) of the M50_75YO_ and F05_75YO_ models where in agreement with the values reported in the literature (Klinich et al., 2012) for the aged population (Table 1).

**TABLE 1:** Comparison of the Bezier angles of the existing models, M50_26YO_ and F05_26YO_, and the newly developed models, M50_75YO_ and F05_75YO_, to the literature data (Klinich et al., 2012).

	Bezier angle (deg)	26 YO model	Young (SD)	75 YO model	Older (SD)
M50	Superior	10.1	10.7 (7)	15.2	18.2 (10.3)
	Inferior	5.3	2.2 (7.3)	16.0	14.7 (12.3)
F05	Superior	15.0	17.1 (11.5)	21.4	24.9 (13.4)
	Inferior	2.2	5.2 (15.6)	14.3	18.1 (12.4)

**FIGURE 6:**
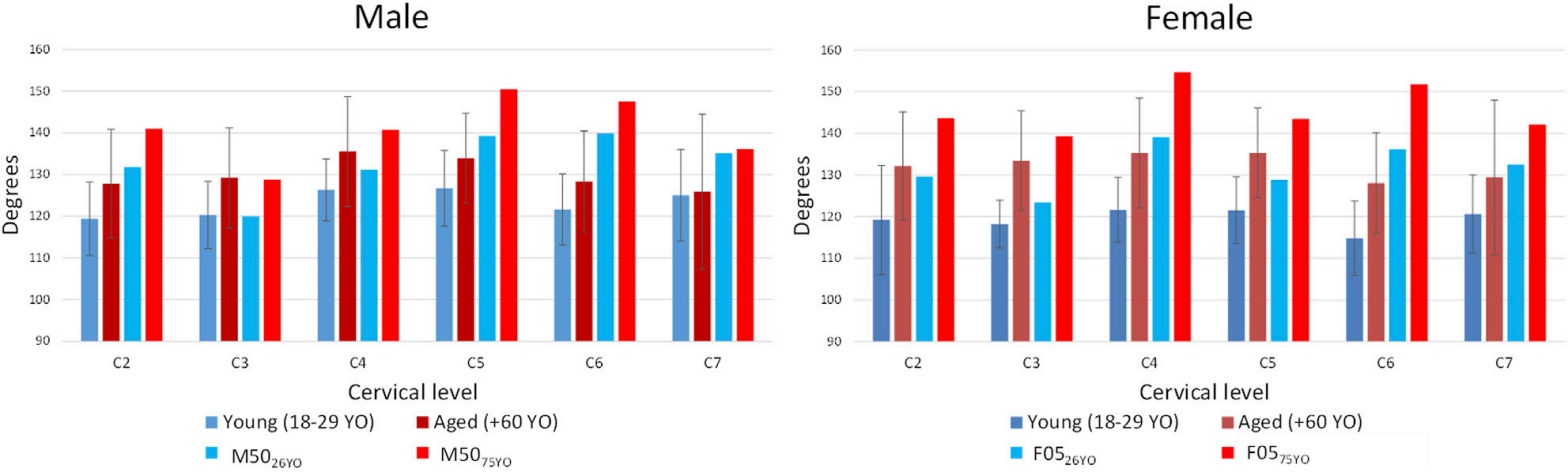
Facet angle change with age reported in the literature and the facet angle of the developed models M50_75YO_ and F05_75YO_.

The facet angle of the M50_75YO_ and F05_75YO_ models (Figure 6) were in agreement with the literature (Parenteau et al., 2014), within one standard deviation of the average with the exception of the C5 and C6 level in the male and C4 and C6 in the female, where the models had a higher facet angle compared to the literature (Figure 6).

### Model Response Assessed With Head Kinematics and Tissue-Level Response

Four models were assessed under six impact conditions (24 analyses in total). The primary head kinematics will be shown together with the experimental data. The non-primary kinematics were monitored as well, but the magnitudes were small and therefore they were not reported in the current study. The FJK and nominal IVD strain was monitored at each segment level. The presented results demonstrate the trends and the effects of impact severity, sex and age for the 8 g frontal and 7 g rear impact cases. In general, the trends observed at the other impact severities (2 and 15 g frontal, 3 and 10 g rear) were similar to those observed at the intermediate impact severities (8 g frontal and 7 g rear). The complete set of results for all impact severities can be found in the supplemental material (Supplementary Appendix A, B, C and D). The head kinematics of the M50_26YO_ and M50_75YO_ under the frontal (2, 8 and 15 g) and rear (3, 7 and 10 g) impacts can be found in Supplementary Appendix A. The FJK and the nominal IVD shear strain at each segment level can be found in Supplementary Appendix B. Similarly, for the F05_26YO_ and F05_75YO_ models, the head kinematic response can be found in Supplementary Appendix C while FJK and the nominal IVD shear strain in Supplementary Appendix D.

### Effect of Impact Severity

**FIGURE 7:**
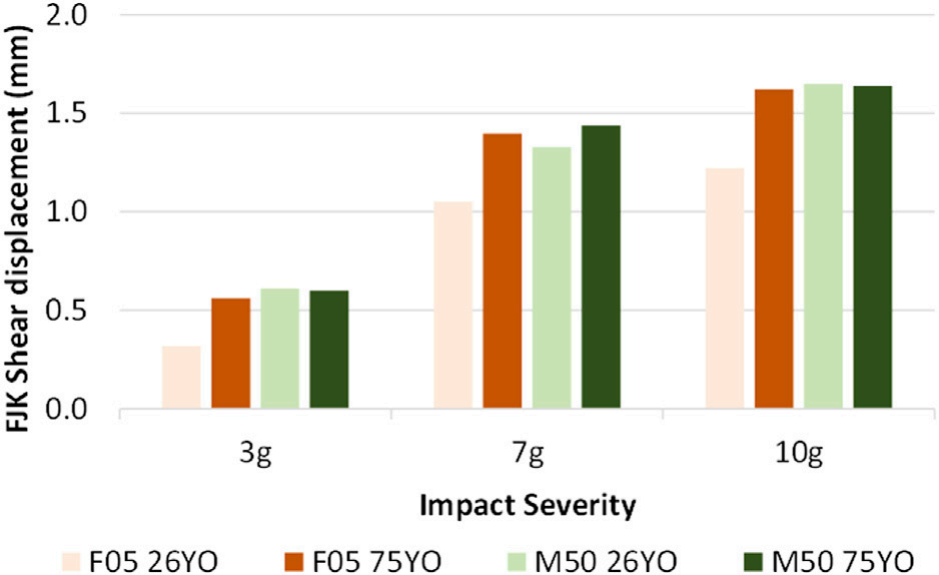
FJK shear displacement, averaged at all segment levels for three rear impact severities, demonstrating increased response with increasing impact severity.

Increasing impact severity led to increases in the magnitude of the head kinematics, FJK, and nominal IVD shear strain, as expected. In that case, in agreement with the epidemiology data severity for the rear impact cases (Figure 7).

### Age Effects

**FIGURE 8:**
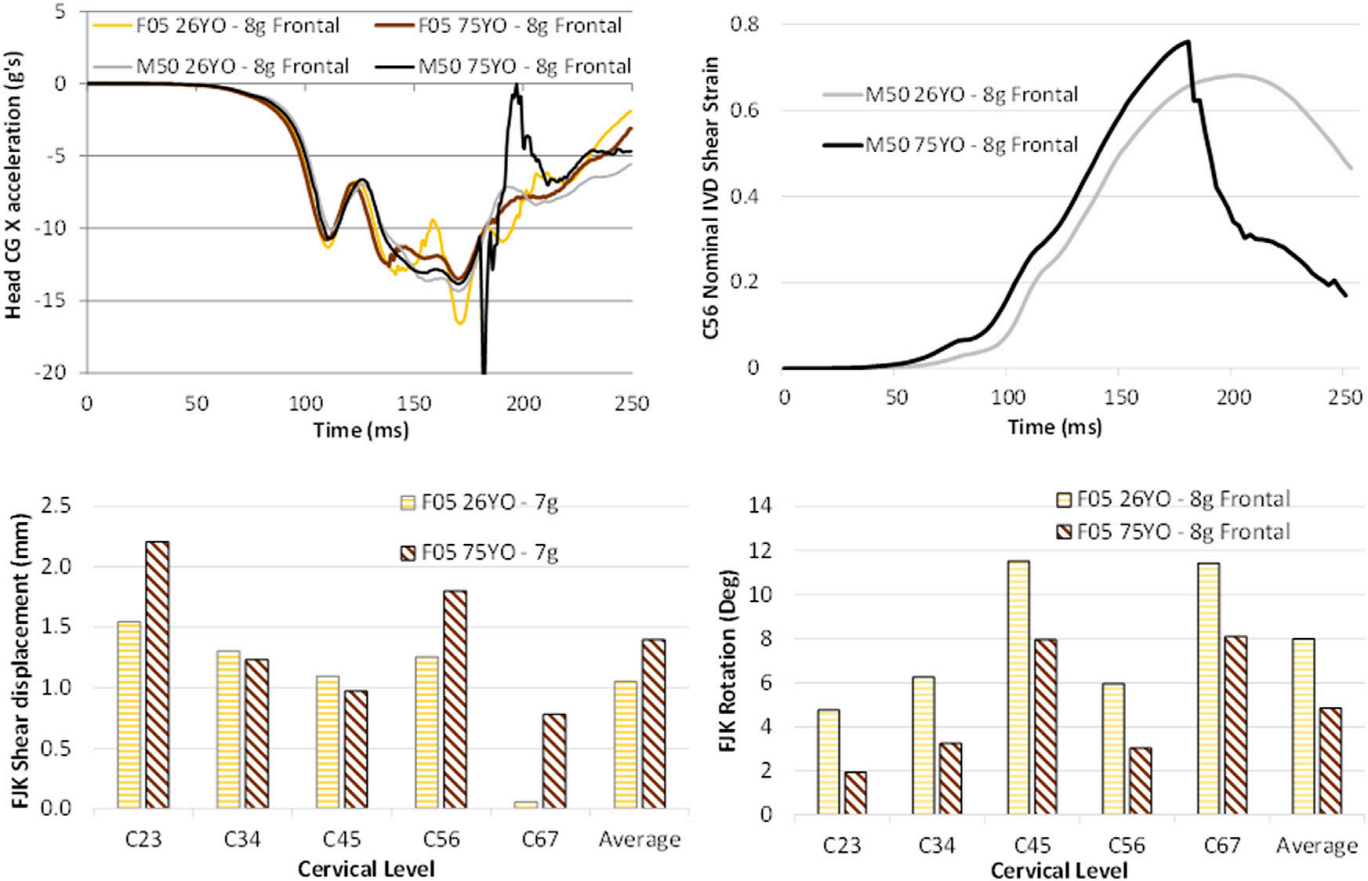
Age effects on the (**A**) head kinematic response of the four assessed models, (**B**) the IVD space shear strain time history for the M50_75YO_ and M50_26YO,_ demonstrating the effect of hard tissue failure, and (**C**) the relative facet joint kinematics in the rear and (**D**) frontal impact demonstrating the age-related differences in the female models.

At the head kinematic level, the young and aged models demonstrated similar head kinematics shapes and peaks compared to the young models (cross-correlation ratings raging from 0.90 to 0.94 suggesting strong correlation). One notable difference was a spike in the head CG linear acceleration in the “X” and “Z” axis and in the rotational acceleration in the “Y” axis for the male model in the 8 g frontal (and 15 g frontal, Supplementary Appendix A and C) due to the hard tissue failure in the 6th vertebra of the M50_75YO_ (Figure 8A). Hard tissue failure occurred only for the M50_75YO_ model in the 8 and 15 g frontal simulations.

The FJK in the male models were similar to one another in the frontal and rear impacts (Supplementary Appendix B). In contrast, for the female models, the F05_75YO_ predicted 26% higher relative facet shear in the rear impact while 36% lower relative facet rotation in the frontal (Figures 8C,D) when compared to the F05_26YO_.

The nominal IVD shear strain between young and aged models was similar for all impact directions and severities, except for the female models in frontal impact (the F05_26YO_ model predicted 17% more strain compared to the F05_75YO_ model) (Supplementary Appendix B and D). However, for the M50_75YO_ 8 g frontal impact, the maximum nominal IVD shear strain was affected by the predicted hard tissue failure. The nominal IVD strain time history demonstrated the maximum value at the moment prior to the hard tissue failure, followed by unloading of the IVD due to the vertebral body fracture (Figure 8B).

### Sex and Size Effects

**FIGURE 9:**
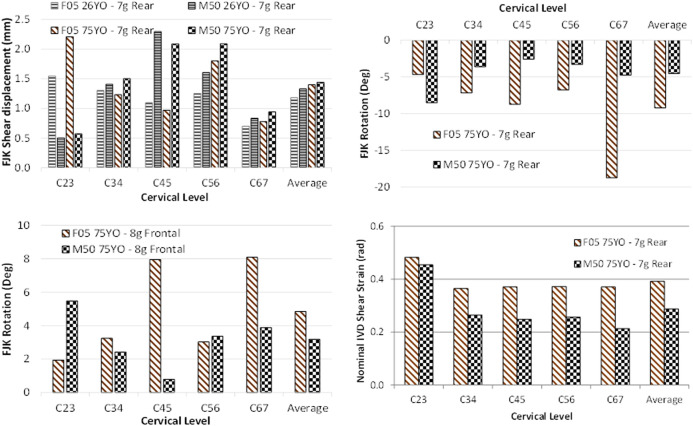
Sex effects in the (**A**) relative facet shear displacement in the rear impact, the (**B**) relative facet rotation in the rear impact, the (**C**) relative facet rotation in the frontal impact, and the (**D**) nominal IVD shear strain in the rear impact demonstrating the differences associated with sex.

The differences between the head kinematic response between the male and female models were modest in general (with cross-correlation ratings ranging from 0.73 to 0.92). One notable difference was a spike in the head CG linear acceleration in the “X” and “Z” axis and in the rotational acceleration in the “Y” axis for the male model in the 8 g frontal (and 15 g frontal, Supplementary Appendix A and C) due to the hard tissue failure in the M50_75YO_ (Figure 8A). Hard tissue failure occurred only for the M50_75YO_ model in the 8 and 15 g frontal simulations. In general, the differences in tissue response observed between the M50_26YO_ and F05_26YO_ were similar in nature to those of the M50_75YO_ compared to the F05_75YO_. For example, male models predicted higher FJK shear displacement (12% more in the young models and 4% more in the aged models) regardless of age in the rear impact (Figure 9A). With respect to the FJK, in the rear impact, the female models predicted double the relative facet rotation on average than that of the male models (Figure 9B). Similarly, for the frontal impacts, the female models predicted 24% more relative facet joint rotation when compared to the male models (Figure 9C). The greater relative facet rotation predicted by the female models in frontal and rear impacts when compared to that of the male model was observed at most segment levels. However, in the C23 segment the facet joint rotation predicted by the male models was higher than that of the female in both in the frontal and in the rear impacts.

In the rear impact, the female model predicted 22% more nominal IVD shear strain on average than the male model (Figure 9D). In the frontal impact, the average nominal IVD shear strain of the male models was similar to that of the female models (Supplementary Appendix B and D) with the exception of the F05_75YO_ that predicted 25% less nominal IVD shear strain than that of the M50_75YO_

## Discussion

The neck models were geometrically aged by changing the curvature and the facet angle. A recent study (Reed and Jones, 2017) indicates that the change in facet joint angle is coupled with the change in spine curvature.

### Reposturing Process

Early in the study, a preliminary assessment of simulation-based methods and a commercial morphing package was undertaken. Important limitations were found in terms of the output mesh quality, the difficulty of defining the boundary conditions for the target posture (for the simulation-based method) and the time-consuming process of defining the transformation rules for the soft tissues (for the morphing method). Although contemporary morphing tools may be able to achieve the same mesh quality as PIPER in the final posture, and can be further improved in efficiency, the open-source nature of the PIPER project allows for repeatability of the process by making the metadata and the software itself available to the community. The metadata used in this study for the F05 was made available through the PIPER web site. Within the PIPER project, metadata for the M50 was already freely available to the community.

### Anthropometry

This study used male and female subject-specific young models repostured to represent average 75 YO subjects. The neck length of the subject-specific male model was higher than the average population reported in the literature. Although the subject selected for the development of the M50_26YO_ model met the average mass and stature requirements, differences in anthropometries at the body region level could vary outside of the average for the target population. Interestingly, the M50_26YO_ FE model neck curvature was straighter than the reported curvature of a 50th percentile 26 YO male, but when accounting for the neck length, the curvature of the M50_26YO_ model was in agreement with the literature (Reed and Jones, 2017). This effect was identified using literature that reports individual vertebral positions. Such information may be obscured when using more general measurements, such as Bezier angles. Such measures depend more on the orientation, position, and shape of C7 and C2, with the mid-level vertebrae positions orientations having a lesser effect on the Bezier angles. Although the comparison of the cervical spine region in the models (male and female, both young and aged) to the FBP show a small discrepancy (within the mean plus two standard deviations), the FBP served to have confidence in the head orientation in a driving posture at the global level. The facet angles of the M50_26YO_ and M50_75YO_ models were within one standard deviation of the reported literature data for males for a given age group. The neck length and facet angles of the female models were within one standard deviation of the reported literature data for females at the given stature and age group.

### Effect of Impact Severity

The effect of the increasing impact severities in frontal and rear impact was intuitive and in agreement with other post-mortem human subjects and anthropomorphic test device experiments (Nie et al., 2016) that predicted higher force peaks with higher impact severities. Higher impact severity led to higher head kinematic peaks, FJK, and nominal IVD shear strain. In the M50_75YO_, the hard tissue failure with increased impact severity, however, led to lower peaks in the nominal IVD shear strain due to the subsequent unloading of the cervical spine as a consequence of the element erosion.

### Age Effects

Higher compressive loads in the vertebral bodies of the M50_75YO_ model, which led to hard tissue failure in the frontal impact, were attributed to the more anteriorly located head CG of the M50_75YO_ when compared to the M50_26YO_ that led to a higher moment-arm generating higher anterior compressive stresses in the vertebrae for frontal impact. Higher compressive loads were observed in the M50_75YO_ model at all segment levels that led to hard tissue fracture at the 6th cervical vertebra within the vertebral body when compared to the M50_26YO_ model. Similarly, higher nominal IVD shear strain was observed in the M50_75YO_ when compared to the M50_26YO_ model.

With respect to the female model, the increased age increased the relative facet shear in the rear impacts while the opposite in the frontal impacts. In the rear impact, the increased lordosis together with the increased facet angle (more horizontally oriented facet joints) of the F05_75YO_ led to a more compliant neck under shear loading as the rotational range of motion was reduced by the change in the relative orientation of the facets. The straighter curvature of the F05_26YO_ and the higher facet angle (more vertically oriented) led to higher relative facet rotation rather than relative facet shear.

The effect of age in the form of increased compressive forces observed in the M50_75YO_ in the frontal impact and increased FJK in the rear impact observed in the F05_75YO_ could imply a higher risk of injury with age for both males and females but related to different tissue-level injuries. Epidemiology shows higher chances of neck injury in the elderly population in general (Lomoschitz et al., 2002; Kahane, 2013) and higher for females in rear impacts (Carlsson, 2012), in agreement with the findings of the present study. Neck curvature and facet angle have demonstrated an effect on the tissue response often associated with injury and pain response (Yoganandan et al., 2001; Cavanaugh, 2006; Quinn and Winkelstein, 2007; Curatolo et al., 2011). Such factors could be important to consider in order to develop more effective safety equipment for the aged population.

### Sex and Size Effects

When comparing the F05 to the M50 models, both young and aged, there were size factors (e.g. stature, neck length and head mass) and sex factors (e.g. facet angle, neck slenderness and neck lordosis). It has been shown that the 5th percentile female is not a simple scaled down geometry from a 50th percentile male (Singh and Cronin, 2017). In addition, it was found that sex differences in features like the facet joint angle were not well predicted using local scale factors, suggesting a complicated relationship between size and sex. A study including 50th percentile male and 50th percentile female would also include both size and sex effects; similarly, including male and female size-matched individuals would include the two effects. Although computational models are a promising tool to isolate the sex effect from the size effect, the aim of the current study was to compare the response of an average male to a small stature female owing to the difference of incidence of injury between these two anthropometry groups. In the rear impact, the female (F05_26YO_ and F05_75YO_) models exhibited higher relative facet rotation and nominal IVD shear strain when compared to the male models (M50_26YO_ and M50_75YO_). The increased FJK and IVD deformation could be attributed to the female neck circumference relative to the length being smaller than in males, as is the vertebral body sizes, than males for size-matched subjects (Vasavada et al., 2008). In addition, the strength of the anterior and posterior muscles has been reported to be lower, 31.5 and 19.0%, respectively, than in males. The modest contribution of the female posterior musculature, when compared to the male in a rear impact, led to a higher sensitivity to geometrical and postural changes in the soft tissue response when compared to the frontal impact, where the posterior musculature is the major contributor. In addition, the increase in lordosis associated with age was higher in the females (2.9 deg increased lordosis when averaging the Bezier angles increase) than in the males (1.2 increased lordosis when averaging the Bezier angles increase) (Reed and Jones, 2017). In consequence, the lordosis of the F05_75YO_ was higher than the lordosis of the M50_75YO_ despite of the F05_26YO_ lordosis being similar to the M50_26YO_ lordosis (Table 1).

This is the first computational study that compares the neck response between an average stature male and a small stature female and between young and aged subjects. However, there were some limitations to the current study.

### Limitations of the Study

Although specific injuries have not been linked to model response in the present study, higher tissue deformations could imply a higher likelihood of injury. In the context of this study, the higher facet joint kinematics could be associated with a higher likelihood of pain response in the facet joint, in that case, in agreement with the epidemiology data that suggest that females are more susceptible to injury in a rear impact. Future work includes the investigation of the injury assessment in the context of detailed HBMs using model tissue kinematics to infer injury risk. Importantly, the present study demonstrated that the assessment at the gross kinematic level might be insufficient to capture the effect of the geometric part of the ageing process. Tissue-level kinematic response assessments, such as FJK, were proved more informative than gross kinematic response, such as head kinematics, and might be required to understand the sex and age effects. To investigate the implication of injury related to the age and sex effects, more work is needed. A relationship between FJK and collagenous fibre realignment of the CL, for example, would be ideal to evaluate the injury risk associated with sex and age in HBMs.

The M50 model has been extensively validated at various levels (motion segment, ligamentous spine, and full neck with active musculature level) for a total of 82 validation cases. However, the F05 model has not been validated as extensively as the M50 has, owning, in part, to the lack of experimental data specific to small stature females. The F05 model was developed after the M50 model and based on a similar methodology used for the M50 model in terms of model and mesh design, material properties, and assessment using experimental data. One limitation of the assessments to date is that many experimental studies either report data for the average stature male, were scaled to represent an average stature male, or, in some cases, did not provide data regarding the subject anthropometric details. For example, the volunteer experimental data used to validate the active response of the M50 full neck model comes, in part, from the Naval Biodynamic Laboratory that performed human volunteer experiments using male military personnel. In a scaling study (Singh and Cronin, 2017), compared the response of the F05 to the M50 at the motion segment level and found that scaling based on the sagittal and transverse plane dimensions was appropriate for these models to compare kinematic response between models. It was noted that scaling did not apply specifically to the facet joint due to the fundamental differences in shape and angle between the male and female vertebrae. Importantly, the F05 validation using the same 82 cases as the M50, indicated a good correspondence to the experimental data, providing confidence in the model results.

The effective plastic strain based cortical and trabecular bone failure criteria should be further validated for the cervical spine under traumatic loading. Currently, the implementation has been validated in the cervical spine for non-catastrophic events, meaning that under the boundary conditions of volunteer human experiments, ligamentous spine experiments, and motion segment level experiments where bone failure was not observed, the model did not predict hard tissue failure. With respect to catastrophic events (Khor et al., 2018), validated the cortical material model in a femur fracture under axial rotation and three-point bending. In the cervical spine (Khor et al., 2017), evaluated the cortical and trabecular bone in the C5-C6-C7 functional spinal unit under axial and eccentric compression. However, the level of validation of the failure criteria, is not at the same level as the general validation of the GHBMC neck model under non-bone-fracture cases.

An important limitation of HBM is the uncertainty that exists with regards to the initial stress state of the modelled tissues in any posture. In a previous cervical motion segment investigation (Boakye-Yiadom and Cronin, 2018), using a C45 segment from the GHBMC M50 model, it was demonstrated that the initial stress state matters in terms of the tissue failure progression; suggesting that the stress state is important for the accurate prediction of the tissue response when considering repositioning. In this study, we aimed to develop neutral posture aged models from existing young neutral posture models. Including the induced strains in the soft tissues of the repostured aged models would have led to an unfair comparison, given that the young models did not account for the initial stress state of the soft tissues as well. Additional work is needed in order to define the stress states of the various tissues commonly modelled in HBM.

The geometric variability in biological tissues is often high. Importantly, the variability in anthropometry greatly increases with age (Parenteau et al., 2014), and it might be a dominant factor in the increased incidence of injury in the aged population. In the present study, geometrical variability was not included. Variability of anthropometry in the ageing process can be challenging to implement in HBMs, partially due to the difficulty of reposturing models to a posture that might largely deviate from the original posture of the model. In addition, the relationship between local geometrical changes associated with age, such as facet angle, and the global changes, such as increased lordosis, is not clear. Subject-specific aged models could help researchers to understand such relationships and to encapsulate the geometrical changes associated with age in a more comprehensive manner; subject-specific modelling is part of future work. It is important to note that in the present study, a small stature female (5th percentile), due to the higher likelihood of injury of this anthropometry group, was compared to a medium-size male (50th percentile). Therefore, the size and sex effects were coupled in the present study. A comparison between the present M50_26YO_ and a recently developed 50th female model (ViVA 50th percentile female model) could provide additional information regarding sex differences. However, the aim of this study was to compare the tissue-level response of an average stature male to that of a small stature female owing to the differences in incidence of injury between these two anthropometry groups. Importantly, in the present study, the material properties of the neck tissues and the muscle activation were not modified so that the known effect of geometric changes with age could be investigated. It is acknowledged that, with increasing age, biological material properties may change and increase in variability, joint stiffness may increase, and hard tissue strength decreases. In the context of the current study, increased joint stiffness may affect the FJK, and the lower strength hard tissue could lead to fractures, both monitored in the present study. Both the change in material properties and the potentially reduced muscle activation force could lead to more tissue distraction and higher injury risk. Including the effect of ageing in the material properties and in the muscle activation scheme is planned for future work. In addition, the boundary conditions applied to the T1 were the same for the four models. It is possible that the T1 response of the different anthropometry groups (young female compared to an aged female) changes under an impact scenario. Additional volunteer experimental data concerning female subjects is needed in order to develop boundary conditions for the neck that are representative of the female response under impact.

The interaction of the aged neck models with the safety systems in a car environment was not studied. Full body studies that compare young and aged models in a car environment or in a sled impact could be more informative about the effect of age on the effectiveness of the safety systems, which is the ultimate goal of the present research path and included in future work.

## Conclusion

In this study, a methodology to modify the cervical spine geometry, using a hybrid approach with CAD and repositioning software (PIPER), successfully achieved the geometric hard tissue targets, while maintaining the overall mesh quality. This methodology could be applied to other models and body regions.

The head kinematic responses in terms of peaks and shape were similar for the four models and a given impact severity. However, the sex and size effects were evident in the tissue-level kinematic responses. Similarly, differences in tissue-level response between the young and aged models were observed and associated with the age-related geometric changes, suggesting that soft tissue metrics could be more informative than gross kinematic response. It is recommended to evaluate soft tissue metrics where possible in computational studies. In addition, detailed measurements of the soft tissue response along with a detailed description of the experimental set-ups in experimental studies would be beneficial for model development and validation at the tissue level. Similarly, when designing safety equipment, it could be more informative to evaluate the soft tissue response in the assessment of the effectiveness of the various safety systems to the protection of the subjects.

The epidemiology suggests that, in rear impacts, small stature female occupants demonstrate an increased risk of WADs when compared to males. This study is supported by those findings in the form of higher FJK predicted by the F05 (both young and aged) when compared to those of the M50 (both young and aged). Therefore, it is important to consider both sexes when evaluating safety systems. Although the importance of considering both females and males has been established before the present study, the present study has identified specific kinematics and could provide guidance for future investigations in injury risk.

The aged models demonstrated, in general, higher tissue deformation than their young counterparts. Higher tissue deformation could be associated with injury, but more work is needed to identify injury thresholds for the various tissues implicated in injury and pain response.

Age, sex and size effects were identified and found to be in general agreement with the existing literature suggesting a higher likelihood of injury for the aged population in general, and in rear impact for the female occupants.

## Data Availability

The original contributions presented in the study are included in the article/Supplementary Material, further inquiries can be directed to the corresponding author.
