# Supplementary material for: Sex, Age and Stature Affects Neck Biomechanical Responses in Frontal and Rear Impacts Assessed Using Finite Element Head and Neck Models
Source: Front Bioeng Biotechnol. 2021 Sep 21;9:681134. doi: 10.3389/fbioe.2021.681134 (PMC8490732; doi:10.3389/fbioe.2021.681134)
Supplement: Supplementary file 4 [file Table3.docx]

# Appendix C: Female models head kinematics.

## Frontal impacts

## 2g

## 8g

## 15g

## Rear impacts

## 3g

## 7g

## 10g
