# Supplementary material for: Sex, Age and Stature Affects Neck Biomechanical Responses in Frontal and Rear Impacts Assessed Using Finite Element Head and Neck Models
Source: Front Bioeng Biotechnol. 2021 Sep 21;9:681134. doi: 10.3389/fbioe.2021.681134 (PMC8490732; doi:10.3389/fbioe.2021.681134)
Supplement: Supplementary file 5 [file Table4.docx]

# Appendix D: Female models FJK and nominal IVD shear strain.

## Frontal impacts

|  |  |
| --- | --- |
|  | |

Figure 1: Relative facet joint kinematics in the frontal impacts for the female models

Figure 2: Nominal IVD shear strain in the frontal impacts for the female models

## Rear impacts

|  |  |
| --- | --- |
|  | |

Figure 3: Relative facet joint kinematics in the rear impacts for the female models

Figure 4: Nominal IVD shear strain in the rear impacts for the female models
